# Challenges in Applying DNA-Binding Protein Predictors to Biological Research

**DOI:** 10.3390/ijms26199785

**Published:** 2025-10-08

**Authors:** Graydon Cowgill, Steven Anthony Strazza, Savannah Wilson, Ranjeeta Odari, Sadia Afrin Bristy, Yongjian Qiu, Sayaka Miura

**Affiliations:** Department of Biology, University of Mississippi, University, MS 38677, USAsstrazza@go.olemiss.edu (S.A.S.); slwilso6@go.olemiss.edu (S.W.);

**Keywords:** DNA-binding prediction, DNA-binding protein, mutation, protein evolution

## Abstract

DNA binding proteins play a crucial role in regulating gene expression, DNA replication, and chromatin organization. While many DNA-binding proteins have been identified, many unique DNA-binding proteins in non-model organisms and recently evolved lineage- or species-specific proteins remain uncharacterized or often lack experimental validation. In addition, genetic variants may alter previously known DNA-binding proteins, leading to loss of binding ability. To address this gap, various computational tools have been developed to predict DNA-binding proteins from protein sequences or structures. Yet, their real-world utility in biological research remains uncertain. To evaluate their effectiveness, we assessed the availability and predictive performance of existing tools using five real-world case studies. We found that most tools were web-based, offering accessibility to researchers without computational expertise. However, many suffered from poor maintenance, including frequent server connection problems, input errors, and long processing times. Among the ten tools that were functional and practical, we found that prediction scores often failed to reflect incorrect outputs, and multiple methods frequently produced the same erroneous predictions. Overall, even a small number of misclassifications can significantly distort biological interpretation, indicating that current DNA-binding prediction tools are not yet sufficiently reliable for empirical research.

## 1. Introduction

Proteins carry out a vast array of essential cellular functions, many of which have evolved through processes such as gene duplication and amino acid substitution [1]. One critical function shared by many proteins is the ability to bind DNA. Approximately 10% of human proteins have this ability [2], which underlies fundamental processes, such as DNA replication, repair, transcriptional regulation, and chromosomal organization [3]. DNA-binding ability is a common feature across many different gene families, each of which may contain multiple members with distinct yet related functions.

An example of this functional diversification is found in the basic Helix-Loop-Helix (bHLH) family of transcription factors [4]. Most bHLH proteins bind DNA through a conserved basic region located adjacent to the HLH dimerization domain and play central roles in regulating gene expression. However, some bHLH members do not have this DNA-binding capability due to key amino acid differences in the basic region [5,6,7,8]. Despite their inability to bind DNA directly, these proteins can still form heterodimers with DNA-binding partners and act as dominant-negative regulators, influencing transcription indirectly.

Beyond evolutionary variation, mutations can disrupt DNA-binding domains and lead to significant biological consequences. For instance, certain mutations in the transcription factor, Forkhead box protein P2 (FOXP2), are associated with speech and language impairments, while alterations in the DNA-binding domain of the tumor suppressor, p53, are commonly linked to cancer [9,10].

Yet, the DNA-binding ability of many proteins and their variants remains uncharacterized across diverse species. To address this, numerous computational tools have been developed to predict DNA-binding ability based on protein sequence or structure [11,12,13,14,15,16,17,18,19,20]. Most benchmarking studies rely on large, curated datasets and emphasize overall accuracy across many proteins, typically reported as the percentage of correctly classified DNA-binding sites. However, even methods with high overall accuracy can be misleading in real-world biological applications. For example, when researchers focus on a small number of uncharacterized proteins, the goal is often to understand the molecular function of specific amino acid residues. Since such scenarios are rarely tested in benchmarking studies, the actual reliability of these methods in typical research contexts remains unclear.

In this study, we therefore assess the practical utility of current DNA-binding prediction tools. Specifically, we ask whether these methods are actively maintained and remain functional, whether they are user-friendly, and whether they provide sufficient reliability. To address these questions, we begin by surveying their availability and usability, and then evaluate their performance in biologically relevant scenarios, including evolutionary comparisons and mutation effect analyses.

## 2. Results

### 2.1. Characteristics of DNA-Binding Prediction Tools

Through an internet search, we identified over 50 computational tools developed to predict the DNA-binding abilities of proteins (Supplementary Material Table S1). Most of these tools were implemented as web-based applications, and tools available exclusively as standalone software were relatively uncommon.

We found that many web-based tools were not maintained well, while a few early tools, such as DP-Bind (2007) and DNABIND (2006) remained functional (Supplementary Material Table S1). The issues included unstable servers or connection failures during data submission or computation. Some tools exhibited these problems consistently, while others were only intermittently affected. To ensure reproducibility, we excluded tools that were unavailable, unstable, or nonfunctional. Also, some others did not clearly output predicted binding status, and we also excluded these tools. In addition, we excluded methods that required extensive computational time, taking more than 6 hours to analyze a single protein.

In total, ten methods met our criteria and were used for further analysis (Table 1). All methods supported sequence-based input, where two of them (NucBind and DNABIND) supported both sequence- and structure-based input. NucBind was relatively slow for both sequence and structure options, requiring a few hours of computation per protein. On the other hand, DNABIND was fast and returned results within seconds. These included five tools that predict DNA-binding residues and five that classify proteins as DNA-binding or non-binding. Four of the ten selected tools were designed to jointly assess DNA- and RNA-binding capabilities. These include DRNApred and NucBind, which predict binding residues, and iDRBP-MMC and iDRPro-SC, which classify proteins into DNA-binding, RNA-binding, or non-binding categories.

The DNA-binding prediction tools evaluated in this study differ in the specific features and strategies they use for classification, though most rely on combinations of physicochemical properties, evolutionary conservation, and/or structural information (Table 1). For example, TargetDNA (residue-level) and iDRPro-SC (protein-level) integrate evolutionary and physicochemical information. TargetDNA additionally uses solvent accessibility features predicted by SANN [21], while iDRPro-SC includes subfunction predictions. DNABIND (protein-level) combines physicochemical and structural features, such as the proportion of specific amino acids (Arg, Lys, Asp, Ala, and Gly), spatial asymmetry of certain residues (Arg, Gly, Asn, Ser), and the protein’s dipole moment. Meanwhile, TargetDBP and hybridDBRpred further incorporate residue-level predictions from external tools.

On the other hand, DP-Bind relied solely on evolutionary features, using position-specific scoring matrices (PSSMs) generated by PSI-BLAST. The strategy of DPP-PseAAC was to exclusively evaluate physicochemical features across several dimensions: (1) dipeptide and (2) tripeptide frequencies, (3) gapped dipeptides, and (4) position-specific combinations of amino acid composition and sequence motifs.

### 2.2. Case Study 1: Prediction of DNA-Binding in the Escherichia coli Lactose Operon Repressor

Advanced sequencing technologies have enabled to sequence bacterial genomes in a few hours, and more than two million bacterial genome sequences are available [22]. Bacterial genome annotations are frequently based on automated computational pipelines, and many bacterial proteins are labeled as “hypothetical protein” [23]. So, many potential DNA-binding proteins remain uncertain without verification.

To evaluate whether DNA-binding prediction tools can reliably distinguish DNA-binding proteins, we selected the well-characterized lactose operon (lac operon) repressor, LacI, from *Escherichia coli*, as a test case. LacI is a transcriptional regulator that binds to the promoter region of the lac operon (comprising *lacZ*, *lacY*, and *lacA*) and inhibits transcription in the absence of lactose. We tested whether computational tools could correctly identify LacI as a DNA-binding protein.

**Figure 1:**
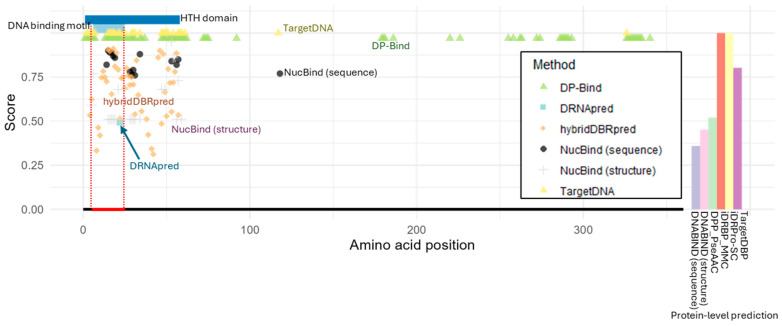
Structure of lactose operon repressor (LacI) and DNA-binding prediction. The helix-turn-helix (HTH) domain and the DNA-binding motif are indicated at the top. The DNA-binding region is marked with a red line at the bottom. For each residue-level prediction method, the predicted probability or score is shown only for residues identified as DNA-binding. For protein-level prediction methods, the bar plot on the right displays the predicted probability or classification score of DNA-binding potential, using the same *y*-axis scale as the plots for the other methods shown on the left. DP-Bind, TargetDNA, and iDRPro-SC generate binary predictions, with DNA-binding classifications indicated by a value of 1.

Figure 1 presents the residue-level DNA-binding predictions from each method. For TargetDNA and DP-Bind, binary classifications were shown, where only residues predicted as DNA-binding are displayed. For the other residue-level prediction tools, the scores or probabilities for residues predicted as DNA-binding are shown.

We found that all tested methods correctly identified DNA-binding residues within the helix-turn-helix (HTH) DNA-binding motif of LacI (Figure 1). Except DRNApred, all tools also predicted many additional DNA-binding residues outside the actual DNA-binding motif but still within the HTH domain, thereby potentially capturing residues that contribute indirectly to DNA binding. For example, the second helix within the HTH motif acts as the recognition helix, binding specifically to the cis-element in the major groove, whereas the first helix primarily interacts with the minor groove to facilitate bending [24].

We next found that NucBind (sequence-based) and TargetDNA made one or a few incorrect predictions outside of the HTH domain, respectively. If a cluster of DNA-binding residues is required as true positives, these errors can be negligible. However, DP-Bind, the oldest tool we tested, went further, incorrectly predicting a large number of false positives across the protein. This pattern supports claims that later tools have been improved for greater accuracy. Although DP-Bind correctly captured key DNA-binding residues, the presence of many false positives outside the functional domain limits their utility for users aiming to discover previously unknown DNA-binding residues. Also, it is important to note that DP-Bind included this protein in its training dataset, as did many other methods (Supplementary Material Table S2). Therefore, the errors observed specifically in DP-Bind do not appear to result from whether this protein was included or excluded in the training dataset.

Protein-level prediction results showed that all tools, except one, correctly identified LacI as a DNA-binding protein, although the prediction score from DPP_PscAAC was relatively low (Figure 1). The only exception was DNABIND, which incorrectly predicted LacI as non-DNA-binding based on both its sequence and structure. Nevertheless, even in this case, the prediction scores (inferred potential of DNA binding) were not extremely low.

While most tools produced accurate classifications, we noticed that a limitation of protein-level methods is their lack of interpretability. Because these methods do not reveal which specific residues or features influence the prediction, it becomes difficult to investigate the source of disagreement or make an informed judgment about which result is more reliable.

### 2.3. Case Study 2: Prediction of DNA-Binding Ability in Mutant Proteins

We next evaluated whether the selected tools could accurately predict DNA-binding ability in mutant proteins. This is a critical task given that the functional impact of most genetic variants remains unknown. In biomedicine, DNA-binding prediction tools could aid in disease risk prediction if they are accurate. To test this application, we examined two well-characterized human transcription factors (FOXP2 and p53).

#### 2.3.1. Forkhead Box P2 (FOXP2)

FOXP2 is a transcription factor known for its critical role in speech and language development [10]. It is primarily expressed in the brain but is also present in the heart, lungs, and digestive system. A missense mutation resulting in an arginine-to-histidine change at position 553 (R553H) has been linked to heritable speech and language disorders [25]. This mutation occurs within the DNA-binding domain and is experimentally known to abolish FOXP2′s DNA-binding ability.

**Figure 2:**
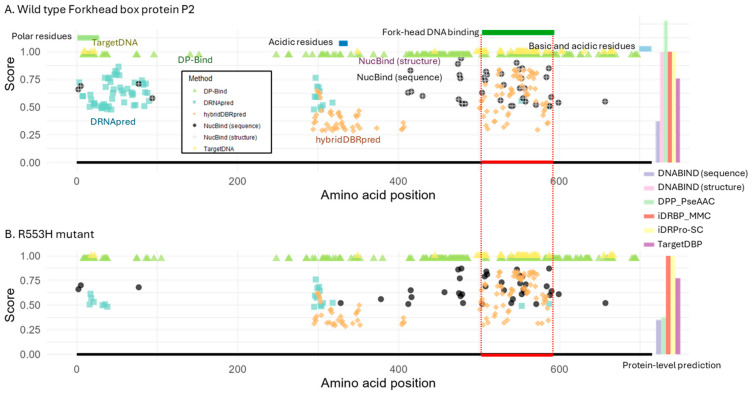
Structure of Forkhead box protein P2 (FOXP2) and DNA-binding predictions. (**A**) Predictions for the wild-type FOXP2 protein. Protein domains and motifs are annotated at the top, and the DNA-binding region is indicated by a red line at the bottom. (**B**) Predictions for the R553H mutant, which is known to lack DNA-binding ability. The red line indicates the DNA-binding region in the wild-type protein for reference. NucBind and DNABIND, with the option of providing protein structures, are excluded from the mutant analysis due to a lack of available protein structure. For residue-level prediction methods, the estimated binding probability or score is shown for each predicted DNA-binding amino acid. For protein-level prediction methods, the bar plot on the right displays the predicted probability or classification score of DNA-binding potential, using the same *y*-axis scale as the plots for the other methods shown on the left. DP-Bind, TargetDNA, and iDRPro-SC provide binary classifications, with a score of 1 indicating a DNA-binding prediction.

We first assessed prediction performance on the wild-type FOXP2 protein (Figure 2A). At the protein level, all tools except DNABIND (sequence-based) correctly identified FOXP2 as a DNA-binding protein with relatively high prediction scores. At the residue level, all tools successfully predicted binding residues within the annotated DNA-binding domain. However, all tools also generated many false positives, predicting DNA-binding residues in regions known not to be involved in DNA interaction. This type of error was also observed in the LacI analysis, but we identified a few different patterns between LacI and FOXP2.

First, while DRNApred’s prediction was accurate on the LacI case (Figure 1), the prediction was not accurate for FOXP2 (Figure 2A). It predicted two large clusters of DNA-binding residues outside the DNA-binding domain. Furthermore, each incorrect cluster contained more residues than the actual binding site itself. We next found that TargetDNA and NucBind (both sequence- and structure-based) produced the fewest false positive detection errors. In the case of hybridDBRpred, it predicted one incorrect cluster, but the associated probabilities tended to be lower than those at the true binding site. So, hybridDBRpred was partially distinguishing correct and spurious predictions.

Overall, the locations of false positives overlapped partially between tools, indicating that a consensus approach would not reliably distinguish true positives from spurious predictions. Additionally, the prediction accuracy varied between FOXP2 and LacI, highlighting that no single method consistently outperforms the others across different proteins.

We next tested the same tools using the mutant FOXP2 sequence (R553H). As the structure for the mutant protein is not available, NucBind and DNABIND were tested only in sequence mode. We found that only DPP-PseAAC and DNABIND correctly predicted the loss of DNA-binding ability in the mutant (Figure 2B). However, DNABIND had also incorrectly predicted that the wild-type did not bind, suggesting that it could not distinguish wild-type from mutant. These results suggested that the tested methods cannot accurately predict the functional impact of mutations on DNA-binding ability, except for DPP-PseAAC.

For the residue-level predictions, many DNA-binding residues were still predicted for the mutant protein by all the methods (Figure 2B). DRNApred and HybridDBRpred showed a slightly smaller number of predicted DNA-binding residues in the mutant compared to wild-type, but still predicted binding residues across the protein, including at the DNA-binding domain. This result seemed to be inconsistent with experimental evidence showing that the mutant protein does not bind DNA [25]. However, a caution is necessary for the interpretation because the loss of DNA-binding activity in mutants can arise through different mechanisms: (1) the mutated residue directly contacts DNA, and its amino acid difference abolishes the interaction; (2) the residue is required for dimerization, which in turn is essential for stable DNA binding; or (3) the mutation alters nuclear localization, preventing DNA access altogether. In the latter two cases, residue-level binding predictions are not informative, since the protein fails to bind DNA for reasons other than the DNA–residue interface itself. In the case of FOXP2 R553H mutant, altered nuclear localization was also reported [26], in addition to the loss of DNA-binding ability without resolved underlying molecular mechanism, i.e., scenario 1 or 2. If the cause is the disrupted dimerization (scenario 2), these prediction methods actually cannot detect the loss of DNA-binding ability due to their design. Similar issues apply species- or lineage-specific loss of DNA-binding ability. To distinguish among these mechanisms, further experimental molecular biology studies are required. Nevertheless, the accumulation and integration of such knowledge into future method development may eventually allow predictions to differentiate the underlying causes of DNA-binding defects. For now, because current prediction methods do not account for these diverse mechanisms, additional assessments are required to reach reliable conclusions.

#### 2.3.2. p53

**Figure 3:**
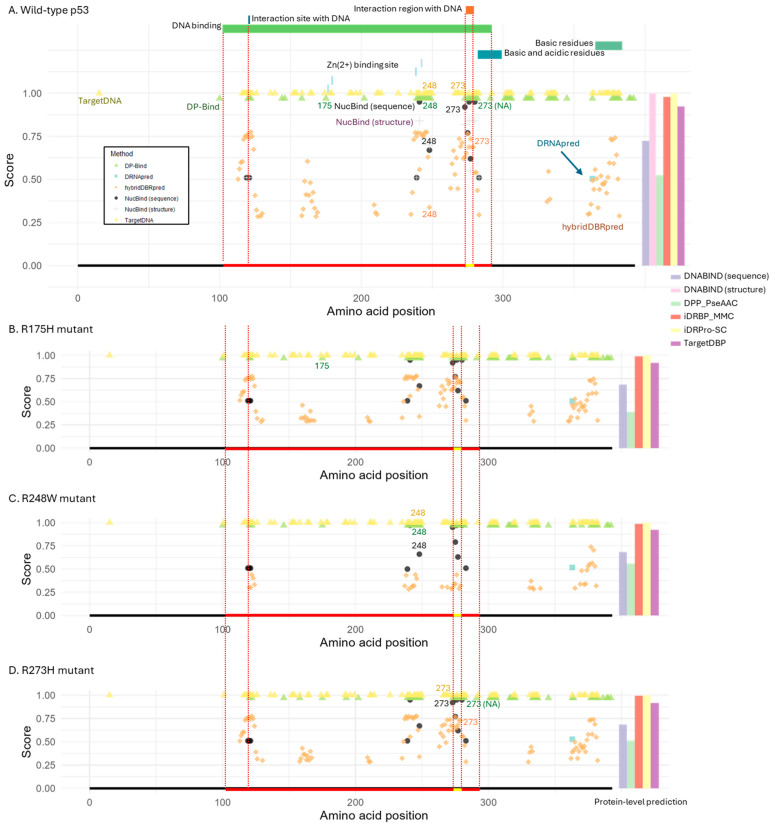
Structure of p53 and DNA-binding prediction. (**A**) Wild-type p53 protein. Functional domains and motifs are annotated at the top, and the known DNA-binding domain is indicated by a red line at the bottom. Two DNA-binding sites are pointed out with a dotted line and a yellow bar within the domain. (**B**–**D**) Cancer-associated p53 mutants: (**B**) R175H, (**C**) R248W, and (**D**) R273H. These mutations impair DNA-binding. For reference, the DNA-binding domain and sites from the wild-type protein are marked by a red and yellow line at the bottom of each panel. For residue-level prediction methods, the estimated probability or score is shown for each predicted DNA-binding residue. For protein-level prediction methods, the bar plot on the right displays the predicted probability or classification score of DNA-binding potential, using the same *y*-axis scale as the plots for the other methods shown on the left. Binary classification methods (DP-Bind, TargetDNA, and iDRPro-SC) use a score of 1 to indicate DNA-binding. Structure-based tools (NucBind and DNABIND) were excluded from panels (**B**–**D**) due to lack of available protein structures for the mutant variants.

Similarly, we evaluated the performance of the prediction tools on p53, a tumor suppressor protein with a well-defined DNA-binding domain (Figure 3A). We focused on three well-characterized, cancer-associated mutations frequently observed in patients, R175H, R248W, and R273H [9]. These mutations occur within the DNA-binding domain and are known to impair p53′s DNA-binding ability. Arg-248 and Arg-273 are DNA-contact residues. In R248W mutation, the introduced large hydrophobic side chain prevent sequence-specific DNA binding [27], while the shorter lateral chains of histidine in R273H compromises the direct contacts to DNA backbone phosphates [9]. On the other hand, a bulky histidine in R175H causes structural distortions, destabilizing the core domain, which lead to the loss of DNA-binding ability [27].

As observed in FOXP2 analysis, most tools detected DNA-binding sites within the DNA-binding domain, but many also predicted additional sites outside the domain in the wild-type p53 protein (Figure 3A). The only exceptions were DRNApred, which predicted a single incorrect site outside the domain, and NucBind, which identified only true positives. Notably, Arg-248 and Arg-273 DNA-contacting sites were correctly predicted as DNA-binding residues by all tools except DRNApred (which missed both) and DP-Bind (which missed Arg-273). It is important to note that this protein, like LacI, was included in most of the methods’ training datasets (Supplementary Material Table S2). Therefore, the performance of these methods may not strongly depend on whether the target proteins are included or excluded from the training datasets.

For the R175H mutant protein, predictions were largely unchanged, likely because this residue does not directly contact DNA (Figure 3B). By contrast, the R248W and R273H mutations occur at residues that directly contact DNA, yet the same positions were still predicted as DNA-binding sites in the mutant proteins by the tools that had detected them in the wild type (Figure 3C,D). The only exception was hybridDBRpred, which correctly detected the loss of DNA-binding ability for R248W.

At the protein level, wild-type and all mutant proteins were consistently classified as DNA-binding (Figure 3A–D). The only exception was DPP-PseAAC, which correctly predicted R175H (structural distortion) as a non-DNA-binding protein. However, the confidence in this prediction (loss of DNA-binding ability) was low, since the score for DNA-binding remained relatively high.

### 2.4. Case Study 3: Applicability to Evolutionary Genomics Studies 

In this case study, we evaluated the utility of DNA-binding prediction tools in the context of evolutionary genomics. Specifically, we asked whether these tools can be used to investigate the evolutionary gain and loss of DNA-binding activity across gene families. Such analyses require knowledge of DNA-binding ability in extant species as a starting point, which is then used to reconstruct ancestral states based on phylogenetic relationships to trace gain and loss events. However, for many species, the information on DNA-binding ability is not experimentally available. In those cases, computational predictions become critical.

As an example, we assessed the performance of available prediction tools using 37 well-characterized bHLH proteins from *Arabidopsis thaliana* (see Section 4). The bHLH gene family is one of the largest transcription factor families and includes both typical DNA-binding proteins [28,29,30,31,32,33,34,35,36] and atypical members that lack DNA-binding activity [37,38,39,40,41,42,43,44,45,46,47].

Large-scale evolutionary analyses typically require batch processing capabilities, but we found that most tools were not designed for this purpose. Among the evaluated methods, only DRNApred (for residue-level predictions) and three protein-level predictors (DNABIND, iDRBP-MMC, and iDRPro-SC) were capable of processing all 37 proteins in a single run (Table 1). Overall, the majority of existing web-based tools are ill-suited for high-throughput evolutionary analyses.

**Figure 4:**
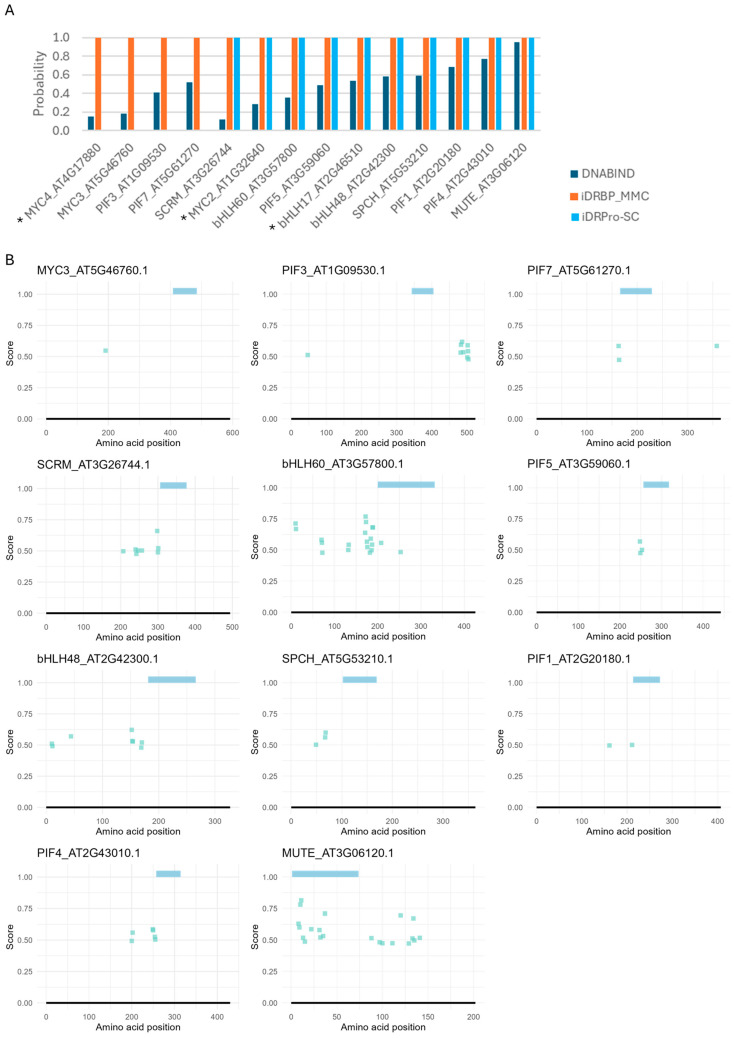
DNA-binding predictions for bHLH proteins with confirmed DNA-binding activity in *Arabidopsis thaliana*. (**A**) Protein-level predictions. DNABIND and iDRBP-MMC provide estimated probabilities of DNA-binding, while iDRPro-SC outputs binary classifications (1 = DNA-binding, 0 = non-DNA-binding). (**B**) Residue-level predictions by DRNApred. Colored bars at the top of each panel indicate the predicted bHLH domain. No DNA-binding residues were predicted for some proteins, which were indicated with * in panel (**A**).

**Figure 5:**
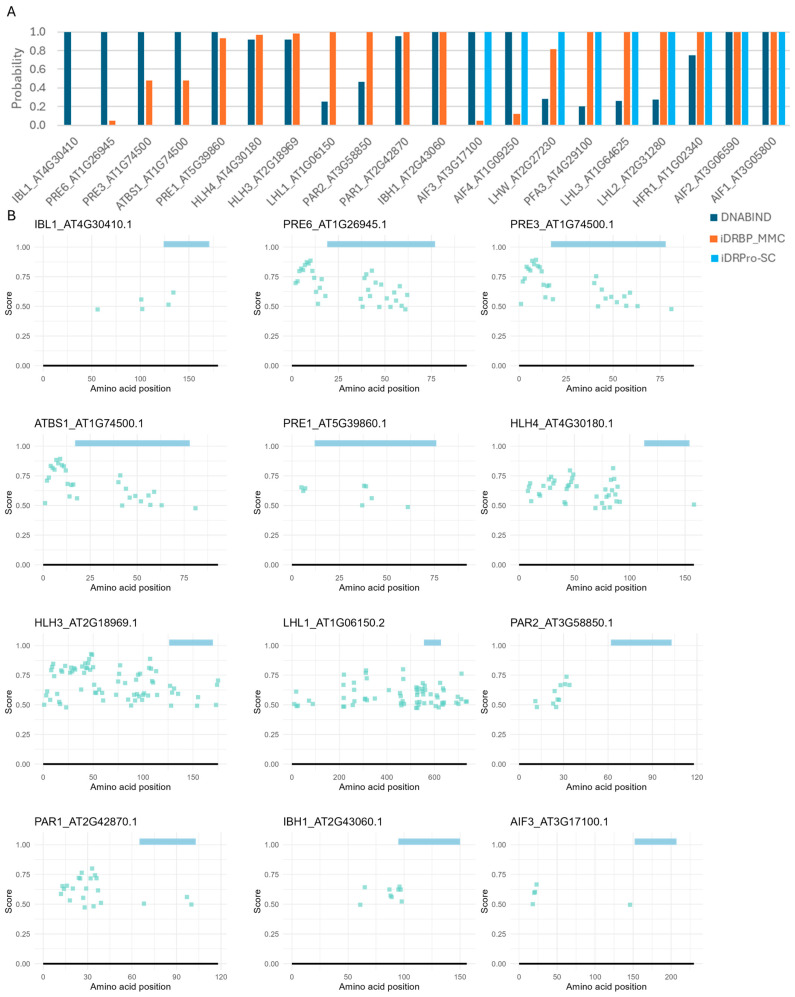
DNA-binding predictions for bHLH proteins lacking DNA-binding activity in *Arabidopsis thaliana*. (**A**) Protein-level predictions. DNABIND and iDRBP-MMC provide estimated probabilities of DNA-binding, while iDRPro-SC outputs binary classifications (1 = DNA-binding, 0 = non-DNA-binding). (**B**) Residue-level predictions by DRNApred. Colored bars at the top of each panel indicate the predicted bHLH domain. The rest of the results are shown in Supplementary Material Figure S1.

We next assessed the performance of the four tools (DRNApred, DNABIND, iDRBP-MMC, and iDRPro-SC) that can handle many proteins in a single run (Figure 4 and Figure 5). For the protein-level predictors, iDRBP-MMC performed well, correctly identifying all DNA-binding proteins (Figure 4A). However, it frequently misclassified non-DNA-binding proteins as DNA-binding, indicating limited specificity (Figure 5A). iDRPro-SC and DNABIND did not perform well in both directions, as they often failed to recognize known DNA-binding proteins while misclassifying non-DNA-binding proteins.

The residue-level predictor (DRNApred) also did not perform well. In bHLH proteins, DNA-binding residues are typically localized within the basic region at the N-terminus of the HLH domain. Additionally, intrinsically disordered regions near the DNA-binding domain may contribute to DNA binding [48,49,50]. While DRNApred correctly predicted one or a few binding residues in this region for many typical (DNA-binding) bHLH proteins, none of DNA-binding sites were predicted for MYC2, MYC3, MYC4, bHLH17, and PIF3 (Figure 4B). For proteins that do not bind DNA (atypical bHLH), one or a few DNA-binding residues at the N-terminus of the HLH domain were often, incorrectly predicted (Figure 5B and Supplementary Material Figure S1). We also tested DNA-binding prediction accuracy for three additional RNA-binding proteins (TAR DNA-binding protein 43, which binds both DNA and RNA [51,52,53], alpha-ketoglutarate-dependent dioxygenase FTO, and cytoplasmic aconitate hydratase [54]) and two proteins known not to bind DNA (Myoglobin and enhancer of zeste homolog 2), including the other prediction methods. While some predictions were correct, these proteins were frequently misclassified as DNA-binding, with many residues incorrectly predicted as DNA-binding sites (Case studies 4 and 5 in Supplementary Note and Supplementary Material Figures S2 and S3).

Overall, the current prediction tools lacked the reliability needed to support evolutionary genomics analyses that depend on accurate inference of DNA-binding residues or DNA-binding capacity in individual proteins.

## 3. Discussion

Our case studies suggest that current DNA-binding prediction tools are poorly suited for biologists who want to use them as part of real data analysis. Even methods that use protein structure information (DNABIND and NucBind) did not perform well. The main problems are that the sources of prediction errors are unclear and the biological reasoning behind each prediction remains inaccessible, making the tools essentially black boxes. Also, it showed no clear or systematic pattern on the prediction across methods and across proteins tested. This inconsistency means that common adjustment strategies—such as consensus predictions or filtering by probability scores—do not offer a dependable solution.

At the same time, it is important to recognize that not all false positives should be dismissed outright. Some predictions may reflect genuine but as-yet undiscovered DNA-binding sites. For example, residues outside canonical DNA-binding domains that are predicted to interact with DNA could represent uncharacterized binding sites. Similarly, proteins previously classified as “non–DNA-binding” may only appear so because they are not normally localized near DNA. If artificially brought into proximity to DNA, they might still interact. From this perspective, prediction methods could be useful for hypothesis generation about unexplored DNA-binding potential. However, such latent binding ability, if never realized under physiological conditions, may have little biological relevance.

On the other hand, our analysis of atypical (non-DNA-binding) bHLH proteins highlights clear-cut errors. These proteins were experimentally demonstrated to lack DNA-binding activity [37,38,39,40,41,42,43,44,45,46,47], yet predictors still flagged binding residues or classified them as DNA-binding proteins. Conversely, for some typical bHLH proteins with validated DNA-binding function [28,29,30,31,32,33,34,35,36], predictors failed to identify binding residues or misclassified them as non-binders.

Taken together, this limited accuracy may be due to the vast diversity of protein–DNA interaction mechanisms [55], making a universal DNA-binding predictor challenging. Therefore, we propose that future efforts should focus on developing gene family–specific prediction models. Within a gene family, members tend to share conserved structural features and binding mechanisms, which should make family-level models more feasible.

Also, it is important to note that many methods intentionally remove similar sequences from their training datasets to avoid bias. As a result, most members of a given gene family are not included in the training data. For instance, in our case study of typical bHLH proteins, none were present in the training datasets of DP-Bind, TargetDNA, or DNABIND. Only MYC2 was included in TargetDBP and iDRBP-MMC (Supplementary Material Table S2). However, bHLH proteins are highly variable, with only the bHLH domain showing some similarity. Moreover, DNA-binding mechanisms often involve regions outside this domain. Global predictors are therefore unlikely to capture subtle differences that influence DNA binding, which can reduce prediction specificity. Overall, we expect that systematically characterizing DNA-binding modes at this gene family-level and incorporating this knowledge could substantially increase specificity.

Our case studies also illustrated that current methods lack the biological reasoning needed for meaningful empirical use. Presenting only a list of predicted DNA-binding residues or a binary classification of proteins as “DNA-binding” or “non–DNA-binding” is superficial, because it provides no insight into how or why binding occurs. Actually, proteins employ diverse mechanisms to interact with DNA: some residues make direct base contacts, while others stabilize the DNA-binding domain through structural support. Direct contacts can be further classified into major or minor groove contact, as well as sequence-specific interaction [55,56]. Similarly, structural support has diverse mechanisms, including dimerization [57]. Furthermore, nuclear localization has an indirect impact for DNA-binding ability [58]. Without incorporating these mechanistic contexts, predictions remain disconnected from the biological reality they are supposed to explain.

Lack of biological reasoning and mechanistic explanation becomes even more pronounced in the context of analysis of the impact of mutations. To interpret the functional impact of a mutation, one must understand the specific role of the affected residue in DNA binding. Mutations can disrupt binding by eliminating direct DNA contacts, destabilizing the structural scaffold, or impairing multimerization interfaces [59]. Each route has different biological consequences. In our case studies, residue-level predictors often reported differences not only at the mutated site but also at distant residues. Such changes could reflect genuine structural consequences. However, without mechanistic reasoning, there is no way to separate meaningful shifts from random false positives.

In conclusion, current DNA-binding protein predictors remain disconnected from biological reality, optimized for benchmark performance rather than for helping biologists interpret experimental data. To become genuinely useful, future methods must not only predict whether a protein binds DNA but also explain how and why. Models that capture mechanistic detail and leverage gene family-specific knowledge should provide deeper biological insight, offering predictions that can directly inform experimental design and hypothesis generation.

## 4. Materials and Methods

### 4.1. Data Assembly

We selected a set of well-characterized proteins with experimentally validated DNA-binding or non-binding functions to illustrate a range of biologically relevant case studies (Table 2). While the selection was random within the pool of annotated proteins, care was taken to ensure that each chosen protein serves as a representative and informative example for our five case studies: (1) DNA-binding prediction in bacterial proteins, (2) prediction of the impact of mutations on DNA-binding ability, (3) assessment of DNA-binding variation within a gene family, (4) evaluation of DNA-binding potential in RNA-binding proteins, and (5) discrimination between DNA-binding proteins and those that bind other molecules or proteins.

Amino acid sequences, together with domain annotations and binding site information, were retrieved from the UniProt database (https://www.uniprot.org/). We also obtained protein structures (PDB files) from the AlphaFold Protein Structure Database (https://alphafold.ebi.ac.uk/). The accession IDs are shown in Table 2.

For FOXP2 and p53, we selected well-characterized, recurrent mutations that are known to affect DNA-binding abilities. These mutations are commonly observed in patients and have been functionally studied in the literature, i.e., R553H for FOXP2 and R175H, R248W, and R273H for p53 [9,25]. Based on the reported mutation sites and corresponding amino acid substitutions, we modified the wild-type protein sequences to generate the respective mutant variants.

For the bHLH protein set, we selected proteins with experimentally validated DNA-binding and non-DNA-binding activities [28,29,30,31,32,33,34,35,36,37,38,39,40,41,42,43,44,45,46,47]. We annotated the bHLH domain using a tool in the InterPro database [60]. NCBI’s Conserved Domain Database (CDD) [61] was used for the annotation.

### 4.2. Selection of Tools and Data Analysis

To identify existing tools for DNA-binding prediction, we conducted a comprehensive web-based search. From the initial list of tools, we retained only those that were functional and accessible at the time of testing. Because most available tools are implemented as web servers, our analysis focused exclusively on methods that offer a web-based interface.

We excluded the tools that met any of the following criteria: (1) the server was consistently or frequently unavailable or non-functional, (2) required more than six hours to process a single protein, or (3) failed to produce outputs or did not clearly annotate DNA-binding ability in the output. Several tools exhibited inconsistent availability, which led us to test them multiple times to assess stability. Any method that proved intermittently accessible or unreliable during the testing period was excluded from further evaluation.

All the tools ultimately selected for analysis accepted amino acid sequences in FASTA format as input (Table 1). Only NucBind and DNABIND also provided the option to input protein structures in PDB format. For all selected tools, prediction results were returned either via email or through a link generated on the web interface. Parameter settings, user options, and output formats for each method are described below.

DP-Bind [14]. Three different encoding options are available to convert a given amino acid sequence into a vector of numerical representation. The first option was (1) Position-Specific Scoring Matrix (PSSM)-based method, which uses the position-specific scores generated by PSI-BLAST (Position-Specific Iterative Basic Local Alignment Search Tool) [62]. Briefly, PSSM represents the degree of evolutionary conservation of each amino acid across homologs of a given protein. The other two available options are based on (2) the evolutionary conservation score derived from BLOSUM62 [63] and (3) sequence-based binary encoding, where each entry in the vector represents one of the 20 amino acids. We selected PSSM because this was the recommended method.

DP-Bind performs three independent predictions, based on the support vector machine (SVM), kernel logistic regression (KLR), and penalized logistic regression (PLR). All three intermediate predictions are produced by DP-Bind. For each prediction, a binary binding label and the probability of DNA binding are produced at each amino acid position in a given protein. DP-Bind’s final prediction is a residue-level binary label of the majority consensus and strict consensus of these three predictions. When a consensus prediction of a given amino acid is not available, these amino acids are labeled as “NA” in the output. For the evaluation, we used the strict consensus.

TargetDNA [17]. For the binary classification of DNA-binding for each amino acid, two strategies to select the thresholds are available, i.e., (1) the threshold that balances the sensitivity (Sen) and specificity (Spe), and (2) the threshold that makes the false positive rate (FPR) to be ~5% (FPR = 1 − Spe). We selected the second option (FPR ≈ 5%), as this was the default option.

For each amino acid, TargetDNA produces a binary binding label and the probability of DNA binding. However, the website does not allow users to download the prediction results, and the table of the probability of DNA binding was not easy to extract from the web interface. Since TargetDNA additionally reports the lists of predicted DNA-binding and non-DNA-binding residues, we used the list of predicted DNA-binding residues for our analysis.

HybridDBRpred [15]. DNA binding residues (DBRs) are predicted by combining predictions generated by three other methods, i.e., DNAgenie [64], DNAPred [65], and DisoRDPbind [66]. For each amino acid in a given protein sequence, hybridDBRpred produces the DNA-binding scores estimated by each method, together with the final combined score and binary classification of DNA binding status. We used the final prediction for our analysis.

DRNApred [12]. DRNApred produces the probability of binding and binary classification (binding ability) for RNA and DNA at each amino acid in a given protein. We used the prediction for DNA-binding for our analysis.

NucBind [18]. Users are allowed to provide a protein sequence or a protein structure. NucBind combines the predictions from SVMnuc and COACH-D. The intermediate inferences from SVMnuc and COACH-D are produced, together with the final inference. Both DNA- and RNA-binding residues are predicted. All inferences include two sets of predicted binding residues, one for DNA-binding and the other for RNA-binding. For each amino acid, the probability of binding and binary classification is given. We used the final prediction for DNA-binding for our analysis.

DNABIND [11]. Similarly, users can provide a protein sequence or a protein structure. DNA-binding ability is predicted using the user-specified threshold of the estimated false-positive rate (FPR). We used the default threshold (FPR equal to 15%). For each protein, the estimated score, probability of DNA binding, and binary classification were produced, which were used for our analysis.

DPP-PseAAC [13]. Chou’s general pseudo amino acid composition (PseAAC) approach [67] is used for this DNA-binding protein prediction (DPP). The score and binary classification of DNA binding are produced for each given protein, which was used for our analysis.

TargetDBP [19]. The probability and binary classification of DNA-binding protein (DBP) are produced for each given protein, which was used for our analysis.

iDRBP_MMC [16]. This method is designed for the identification of DNA- and RNA-binding proteins (iDRBP). It is based on a multi-label learning model and motif-based convolutional neural network (MMC). For a given protein, DNA-binding probability, DNA-binding binary classification, RNA-binding probability, and RNA-binding binary classification are produced. We used DNA-binding probability for our analysis.

iDRPro-SC [20]. This method is for identifying DNA- and RNA-binding proteins (iDRPro) based on subfunction classifiers (SC) of a protein. Only binary classification is produced, which was used for our analysis.

### 4.3. Assessments of Prediction Tools

For methods that generate a score or probability of DNA-binding for each amino acid residue, we evaluated prediction accuracy using only the scores or probabilities assigned to residues classified as DNA-binding. For methods that provide predictions at the protein level, we assessed whether each protein was correctly classified as DNA-binding or non-DNA-binding.

## Figures and Tables

**Table 1:** DNA binding prediction tools tested in this study.

	Residue					Protein				
Method	DP-Bind	TargetDNA	HybridDBRpred	DRNApred +	NucBind *+	DNABIND *	DPP-PseAAC	TargetDBP	iDRBP-MMC +	iDRPro-SC +
Link	https://lcg.rit.albany.edu/dp-bind/ (accessed on 8 September 2025)	https://csbioinformatics.njust.edu.cn/TargetDNA/ (accessed on 8 September 2025)	https://biomine.cs.vcu.edu/servers/hybridDBRpred/ (accessed on 8 September 2025)	https://biomine.cs.vcu.edu/servers/DRNApred/ (accessed on 8 September 2025)	https://yanglab.qd.sdu.edu.cn/NucBind/ (accessed on 8 September 2025)	https://dnabind.szialab.org/ (accessed on 8 September 2025)	http://77.68.43.135:8080/DPP-PseAAC/ (accessed on 8 September 2025)	https://csbioinformatics.njust.edu.cn/targetdbp/ (accessed on 8 September 2025)	http://bliulab.net/iDRBP_MMC/server (accessed on 8 September 2025)	http://bliulab.net/iDRPro-SC/server (accessed on 8 September 2025)
Publication year	2007	2017	2024	2017	2019	2006	2018	2020	2020	2023
Physicochemical property	NA	SA (SANN)	AA (polarizability, charge, hydrophilicity, propensity for intrinsic disorder), SA (ASAquick)	SA (PROFphd, NETASA, and RVP-net)	NA	Proportion of Arg, Lys, Asp, Ala, and Gly	AA (frequency)	AA (frequency), pseSA (SANN)	Protein motif	Protein subfunction (bi-LSTM)
Protein structure	NA	NA	Disorder (IUPred3)	SS (PSIPRED), Disorder (IUPred and Espritz)	SS (PSIPRED), SM (HHblits)	Spatial asymmetry of Arg, Gly, Asn, and Ser; Dipole moment	NA	NA	Protein structual motif	NA
Evolutionary information	PSSM (PSI-BLAST)	PSSM (PSI-BLAST)	NA	EP (HHblits)	PSSM (PSI-BLAST)	NA	NA	psePSSM (PSI-BLAST)	PSSM (PSI-BLAST)	PSSM (PSI-BLAST)
DBR prediction of other methods	NA	NA	DNAPred, DNAgenie, and DisoRDPbind	NA	SVMnuc and COACH-D	NA	NA	TargetDNA	NA	NA
Maximum number of proteins per run	1	1	1	>=37	1	>=37	1	5	>=37	>=37

* Option for providing protein structure. + Prediction for both DNA and RNA binding residues or proteins. AA: amino acid, SA: solvent accessibility, pse: pseudo, SS: Secondary structure, SM: structure model, PSSM: Position-Specific Scoring Matrix, DBR: DNA binding residues.

**Table 2:** Proteins used in this study.

Protein	UniProID	PDB ID
***DNA-binding protein***		
Lactose operon repressor (LacI)	P03023	LACI_AF-P03023-F1-model_v4
Forkhead box protein P2 (FOXP2)	O15409	2A07_AF-O15409-F1-model_v4
p53	P04637	P53_AF-P04637-F1-model_v4
TAR DNA-binding protein 43 (TDP-43)	Q13148	7Q3U_AF-Q13148-F1-model_v4
***Non-DNA-binding protein***		
Cytoplasmic aconitate hydratase (Aconitase)	P21399	2B3X_AF-P21399-F1-model_v4
Enhancer of zeste homolog 2 (EZH2)	Q15910	4MI5_AF-Q15910-F1-model_v4
Myoglobin	P02185	1MBN_AF-P02185-F1-model_v4
Fat mass and obesity-associated protein (FTO)	Q9C0B1	7CKK_AF-Q9C0B1-F1-model_v4

## Data Availability

No new data were created.

## Supplementary Materials

The following supporting information can be downloaded at: https://www.mdpi.com/article/10.3390/ijms26199785/s1.
