# Supplementary material for: Challenges in Applying DNA-Binding Protein Predictors to Biological Research
Source: Int J Mol Sci. 2025 Oct 8;26(19):9785. doi: 10.3390/ijms26199785 (PMC12524727; doi:10.3390/ijms26199785)
Supplement: Supplementary file 1 [file ijms-26-09785-s001.zip › Supplementary material.pdf]

Supplementary Figure

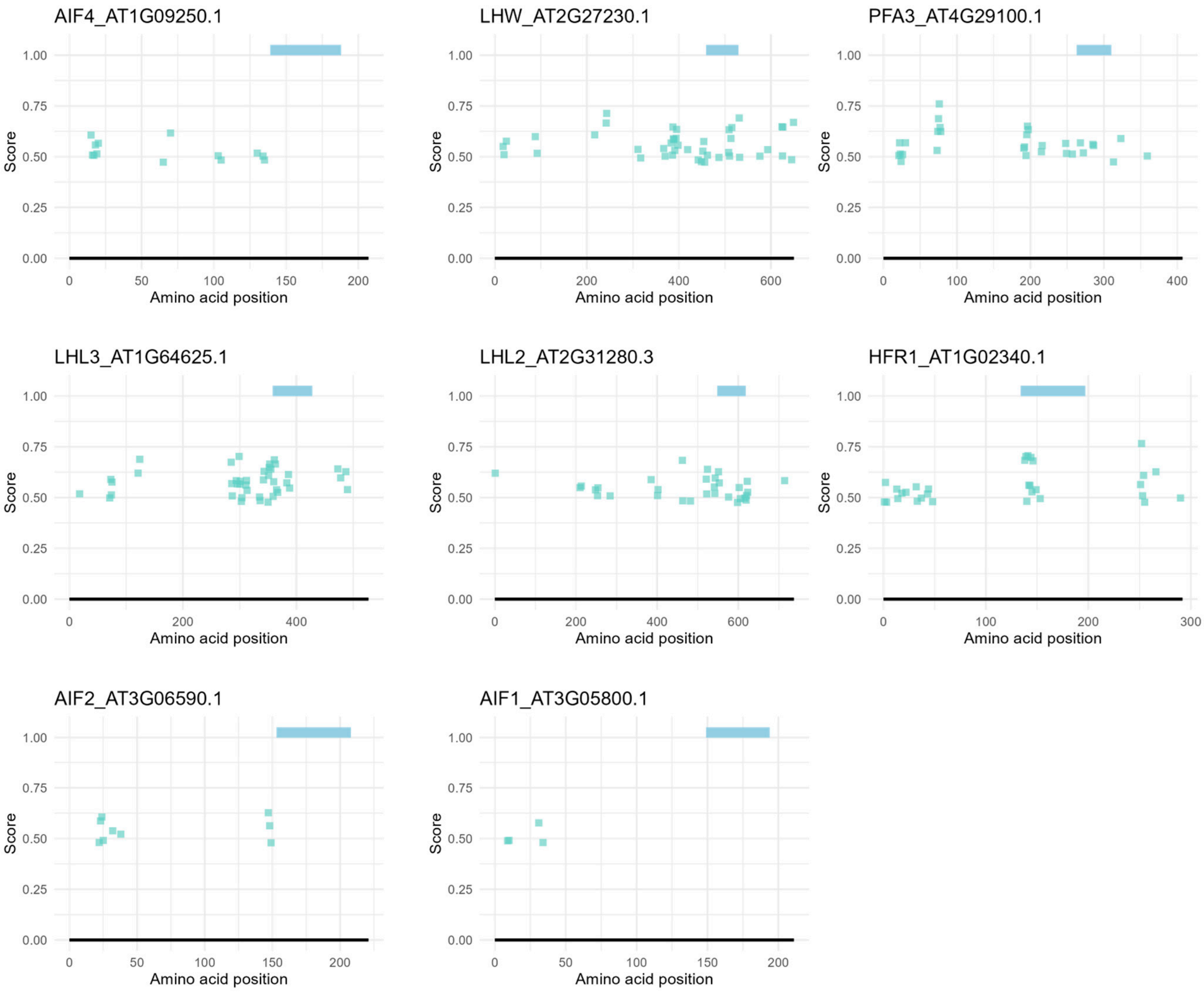

Figure S1. Residue-level predictions for bHLH proteins lacking DNA-binding activity in *Arabidopsis thaliana*. DRNApred was used. Colored bars at the top of each panel indicate the predicted bHLH domain.

Supplementary Tables

Table S1: Prediction tools tested. Excel file was attached.

Table S2: Inclusion of proteins in training datasets across methods.

Protrein	DP-Bind	TargetDNA	DNABIND	TargetDBP	iDRBP-MMC
Lactose operon repressor	1CJG	1EFA	1EFA	1LBG	P03023
Forkhead box protein P2		2AS5			
P53	1TSR	4MZR	1TSR	4MZR, 1KZY	P04637
TAR DNA-binding protein 43		4IUF		5MRG	
PIF1					
PIF3					
PIF4					
PIF5					
PIF7					
SPCH					
MUTE					
SCRM					
bHLH48					
bHLH60					
MYC2				5GNJ	Q39204
MYC3					
MYC4					
bHLH17					

iDRPro-SC, hybridDBRpred, DRNApred, NucBind, and DPP-PseAAC were excluded because these methods used their own protein identifiers, which could not be mapped to standard protein IDs. For proteins included in the training datasets, the corresponding PDB or UniProt ID is provided. Methods and proteins highlighted in yellow were used in our case study.

Supplementary Note

Case Study 4: Prediction accuracy for RNA-binding proteins

Distinguishing DNA-binding residues from RNA-binding residues remains a significant challenge in computational biology [1]. Although models trained on DNA- and RNA-binding information should capture features specific to each type, the fact that some RNA-binding proteins could bind single-stranded DNA (ssDNA) as well may complicate the generation of an accurate prediction model. To explore this further, we evaluated the performance of the prediction methods on RNA-binding proteins.

Transactive Response (TAR) DNA-Binding Protein 43 (TDP-43)

TDP-43 possess both DNA- and RNA-binding capabilities. Initially discovered for its ability to bind the TAR DNA element of HIV-1 and repress transcription [51], TDP-43 is now recognized as a key regulator of RNA biosynthesis and processing [52,53]. It contains two RNA recognition motifs, RRM1 (residues 104–200) and RRM2 (191–262) (Figure 6A). Notably, RRM2 can also bind short DNA fragments.

Our analysis showed that most residue-level predictors correctly identified DNA-binding residues within RRM2, with the exception of DRNApred (Figure 6A). However, consistent with trends observed in the other proteins, all methods also produced false-positive predictions outside of RRM2. These included predictions within RRM1, a domain known to bind RNA rather than DNA. Notably, NucBind is capable of predicting both DNA- and RNA-binding residues but misclassified several RNA-binding residues in RRM1 as DNA-binding sites. Except for hybridDBRpred, all methods also predicted DNA-binding residues in regions that are neither associated with DNA nor RNA binding. These results suggested that misclassifications arise not only from confusion between RNA- and DNA-binding signals but also from broader inaccuracies in identifying functionally relevant binding regions.

At the protein level, two methods (iDRBP-MMC and iDRPro-SC) explicitly model both DNA- and RNA-binding potential. iDRBP-MMC predicted high RNA-binding probability (~100%) and low DNA-binding probability (~14%). iDRPro-SC correctly labeled the protein as RNA-binding but cannot additionally evaluate DNA-binding potentials. Other protein-level methods are designed to assess DNA-binding only. DPP_PseAAC and TargetDBP both predicted that TDP-43 is a DNA-binding protein with high confidences. DNABIND predicted no DNA-binding when using the protein sequence, but predicted DNA-binding when using structural input. Because these methods do not provide a rationale for their predictions, it is unclear whether they misinterpreted RNA-binding signals as DNA-binding or correctly captured the DNA-binding potential. These limitations reduce their utility in assessing proteins with dual DNA/RNA-binding functions.

Fat Mass and Obesity-Associated Protein (FTO)

We next analyzed a protein that binds exclusively to RNA rather than DNA. As an example, we examined FTO, which is a member of the superfamily of alpha-ketoglutarate-dependent hydroxylase. FTO demethylates RNA, and three regions interact with RNAs (Figure 6B).

We found that all residue-level predictors incorrectly assigned DNA-binding residues at one or more RNA-binding sites, including those designed to assess both DNA- and RNA-binding (NucBind and DRNApred) (Figure 6B). Notably, DRNApred failed to correctly identify any RNA-binding residues and misclassified key RNA-binding regions as DNA-binding. NucBind produced similar errors.

At the protein level, DPP_PseAAC and iDRBP-MMC correctly identified FTO as a non-DNA-binding protein, with iDRBP-MMC further classifying it as RNA-binding. In contrast, TargetDBP predicted weak DNA-binding potential (~53% confidence), while iDRPro-SC and DNABIND misclassified FTO as a DNA-binding protein.

Cytoplasmic Aconitate Hydratase (Aconitase)

We also analyzed another RNA-binding protein, aconitase, where RNA-binding activities are context-dependent. When iron is low, it binds mRNAs to regulate the expression of genes that are involved in iron uptake (Figure 6C) [54]. Under high iron, it interacts with a 4Fe-4S cluster and exhibits aconitase activity, inhibiting RNA binding.

For this protein, hybridDBRpred correctly predicted no DNA-binding residues, and DRNApred incorrectly identified only a single DNA-binding residue (Figure 6C). However, the other methods incorrectly predicted DNA-binding residues across the protein. On the other hand, all protein-level predictors correctly classified aconitase as a non-DNA-binding protein. iDRBP-MMC also accurately identified its RNA-binding capability, whereas iDRPro-SC failed to recognize this function.

Overall, prediction performance varied depending on the specific protein being analyzed. Also, false-positives across the proteins suggest that prediction errors are not solely due to confusion with RNA-binding sites.

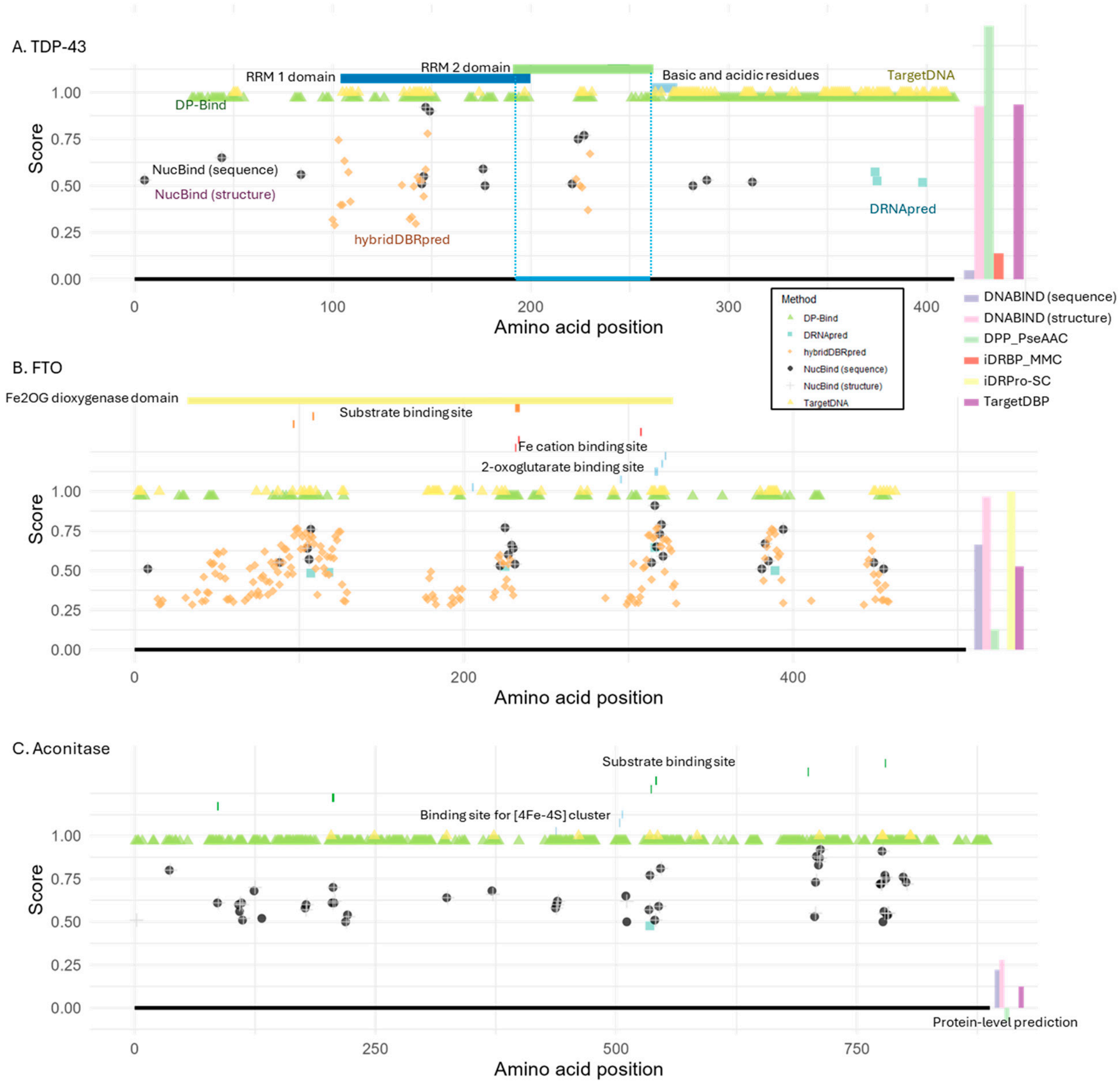

Figure S2. Prediction results for RNA-binding proteins. (**A**) TAR DNA-binding protein 43 (TDP-43), which binds both DNA and RNA. (**B**) Alpha-ketoglutarate-dependent dioxygenase FTO, which binds only RNA. (**C**) Cytoplasmic aconitate hydratase (Aconitase), which is a context-dependent RNA-binding protein. Protein domains and functional motifs are indicated at the top of each panel. DNA-binding domain is shown in blue line. For each residue-level prediction method, the predicted probability or score for DNA-binding is shown for each amino acid classified as DNA-binding. For protein-level prediction methods, the bar plot on the right displays the predicted probability or classification score of DNA-binding potential, using the same y-axis scale as the plots for the other methods shown on the left. DP-Bind, TargetDNA, and iDRPro-SC provide binary predictions, with a value of 1 indicating classification as a DNA-binding protein.

Case study 5: Discrimination between DNA-binding proteins and those that bind other molecules or proteins

We evaluated whether the prediction methods could accurately identify proteins that are known not to bind DNA or RNA. Methods that accurately exclude non-binders can help narrow down the list of proteins with unknown functions to those most likely to have DNA-binding activity.

Myoglobin

First, we examined myoglobin from the sperm whale. Myoglobin functions as an oxygen storage protein, facilitating oxygen diffusion into tissues. It consists of a single globin domain, which spans most of the protein. Within this domain, there are two well-characterized ligand-binding sites: oxygen and nitrite bind at residue 65, and the site for the iron of heme b at residue 94 (**Fig. S2A**).

Several methods correctly identified myoglobin as a non-DNA-binding protein, i.e., iDRPro-SC, iDRBP-MMC, TargetDBP, DNABIND (sequence), and NucBind (**Fig. S2A**). While DNABIND (structure) and DPP_PseAAC incorrectly predicted DNA-binding function, their confidence level was relatively low (~60% to be DNA-binding protein). However, TargetDNA, DP-Bind, DRNApred, and hybridDBRpred incorrectly predicted DNA-binding residues across the protein.

Enhancer of zeste homolog 2 (EZH2)

We also analyzed a protein known to interact with many other proteins, EZH2, which is a histone-lysine N-methyltransferase enzyme. EZH2 is an enzymatic component of the Polycomb Repressive Complex 2 (PRC2). PRC2 includes EED (embryonic ectoderm development), which interacts with EZH2 (**Fig. S2B**). The PRC2 complex may also interact with DNA methyltransferases (DNMT1, DNMT3A, and DNMT3B) via EZH2. EZH2 additionally interacts with the chromodomain Y-like protein (CDYL).

Compared to myoglobin, all methods did not perform well on EZH2 (**Fig. S2B**). DPP_PseAAC and TargetDBP correctly classified EZH2 as a non-DNA-binding protein; however, their confidence scores were only slightly below 50%, i.e., low certainty. The other protein-level prediction methods incorrectly detected it as a DNA-binding protein with relatively high confidence. While NucBind made no false predictions on myoglobin, it incorrectly identified DNA-binding residues in EZH2. In contrast, DRNApred showed better performance on EZH2 than on myoglobin, with fewer false positives, though still not perfect. Similarly, the other residue-level prediction methods incorrectly predicted DNA-binding residues across the protein.

Overall, these tools were also not well-suited for identifying proteins that lack DNA-binding and RNA-binding activity.

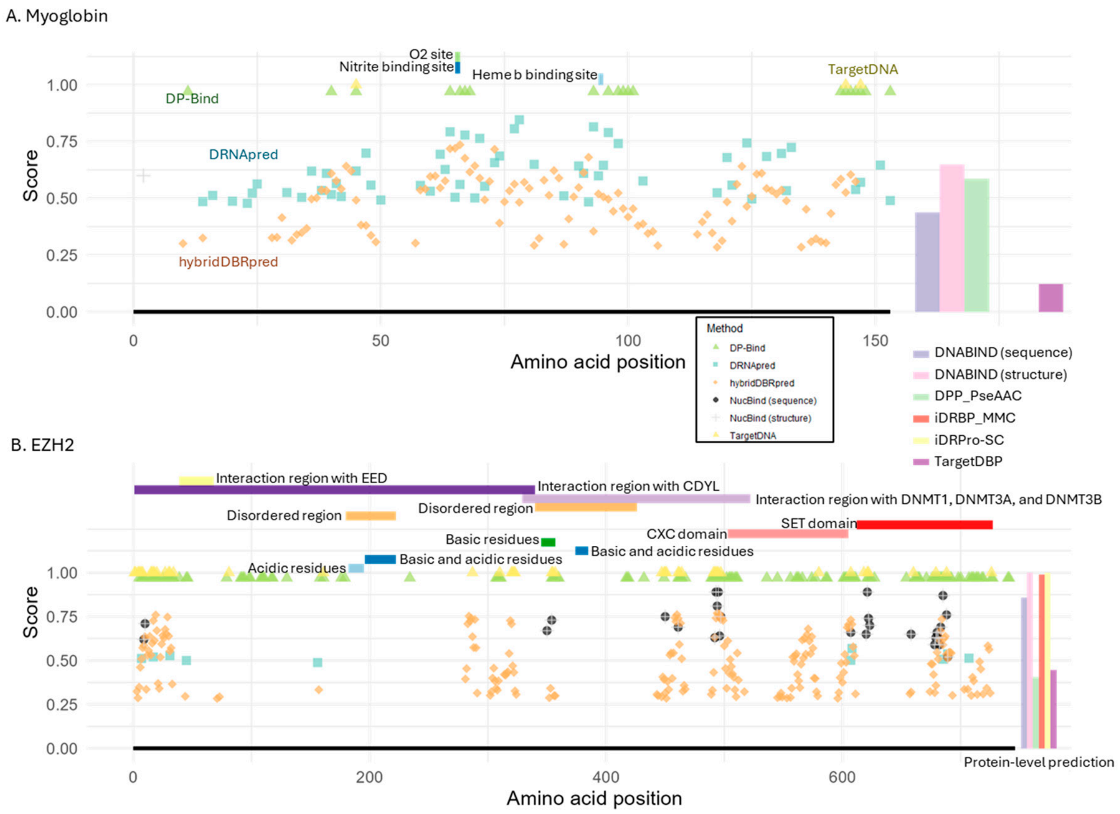

Figure S3. Prediction results for proteins that do not bind DNA nor RNA. (A) Structure and prediction results for myoglobin. Binding sites for nitrite and oxygen are shown with purpose lines. (**B**) Structure and prediction results for histone-lysine N-methyltransferase EZH2. Domains and motifs are shown at the top of each panel. For EZH2, key interacting partners include DNA methyltransferases (DNMTs), embryonic ectoderm development protein (EED), and chromodomain Y-like protein (CDYL). EZH2 contains a SET domain [Su(var)3-9, Enhancer-of-zeste, and Trithorax] and a CXC domain composed of three C-X(6)-C-X(3)-C-X-C motifs. For each residue-level prediction method, the predicted DNA-binding residues are shown along with their corresponding probabilities or scores. For protein-level prediction methods, the bar plot on the right displays the predicted probability or classification score of DNA-binding potential, using the same y-axis scale as the plots for the other methods shown on the left. DP-Bind, TargetDNA, and iDRPro-SC produce binary outputs, with DNA-binding classification indicated by a value of one.
